# Supplementary figures and images for: Prioritizing interventions for preventing COVID-19 outbreaks in military basic training
Source: PLoS Comput Biol. 2022 Oct 7;18(10):e1010489. doi: 10.1371/journal.pcbi.1010489 (PMC9581358; doi:10.1371/journal.pcbi.1010489)

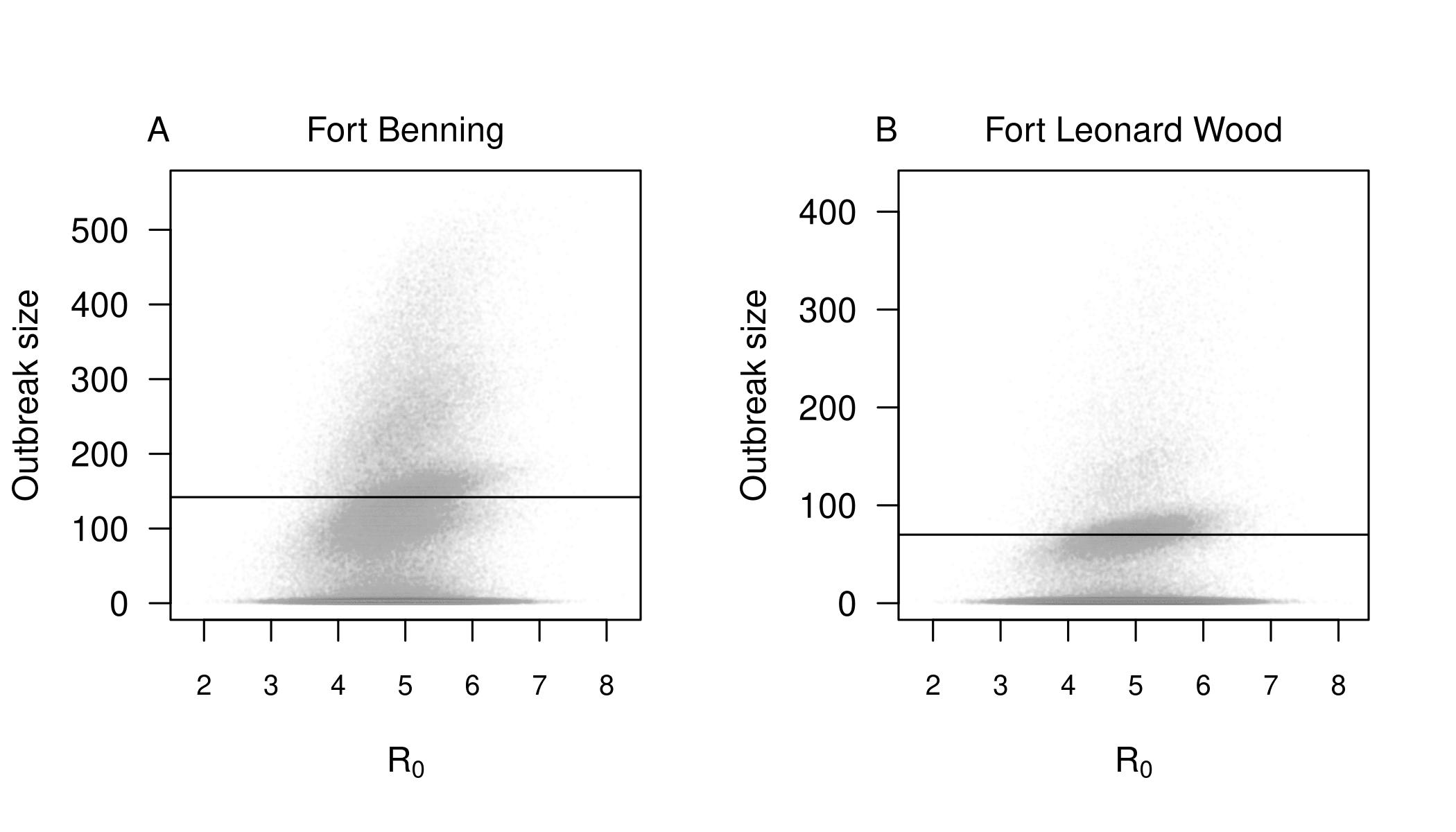

Supplement: S1 Fig — We simulated 200,000 replicate outbreaks for values of R0 evenly spaced between 2 and 20. The horizontal line shows the observed data. (TIF) [file pcbi.1010489.s001.tif]

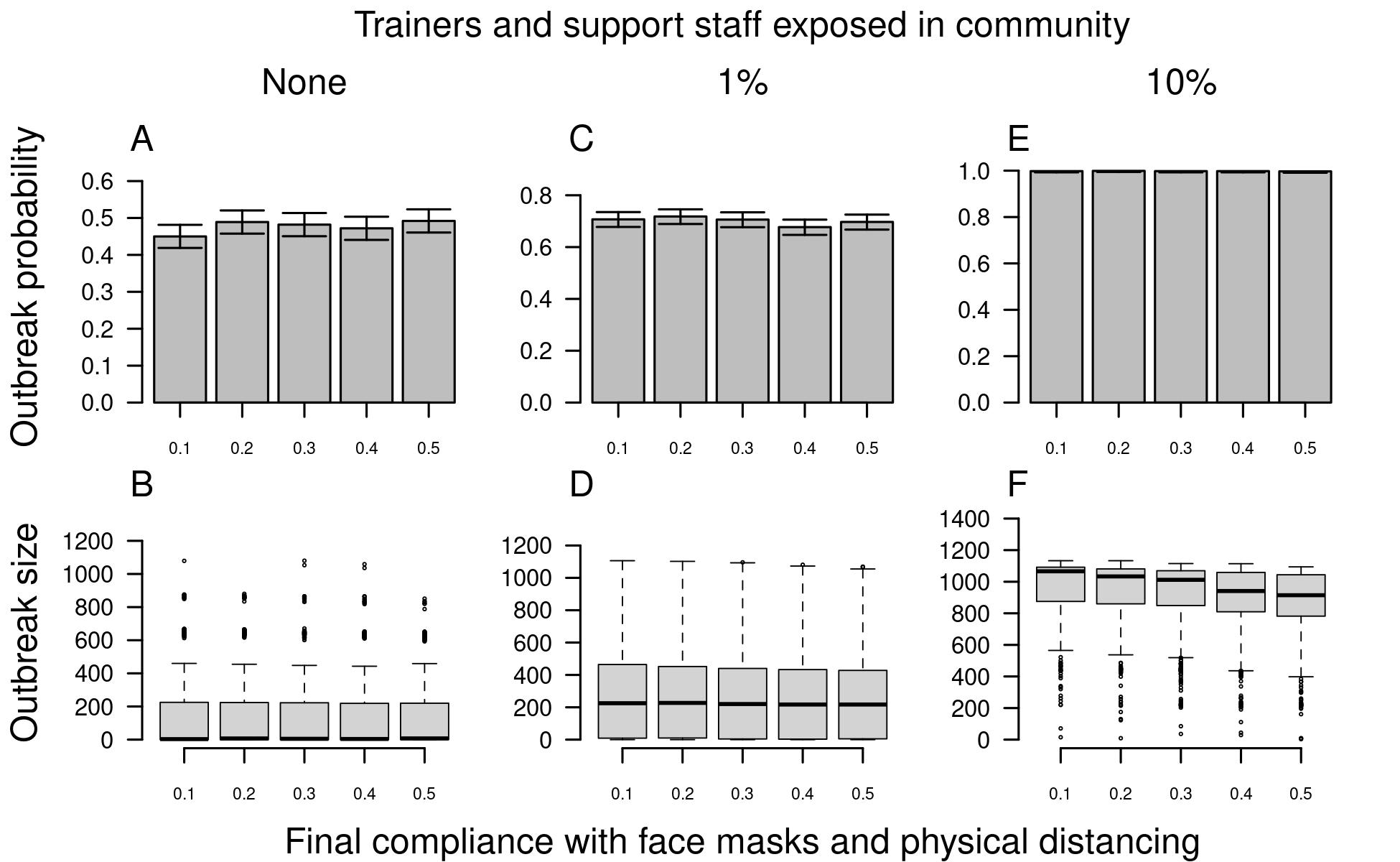

Supplement: S2 Fig — The starting proportion of compliance was set to the baseline value of 0.3, which linearly increased or decreased over time to its final value by the end of the training period. From left to right, columns show increases from 0 to 0.01 to 0.10 of the probability that trainers and support staff were exposed to the virus in the community over the course of the 70-day training period. Error bars for outbreak probability indicate 95% Pearson-Clopper confidence intervals. (TIF) [file pcbi.1010489.s002.tif]

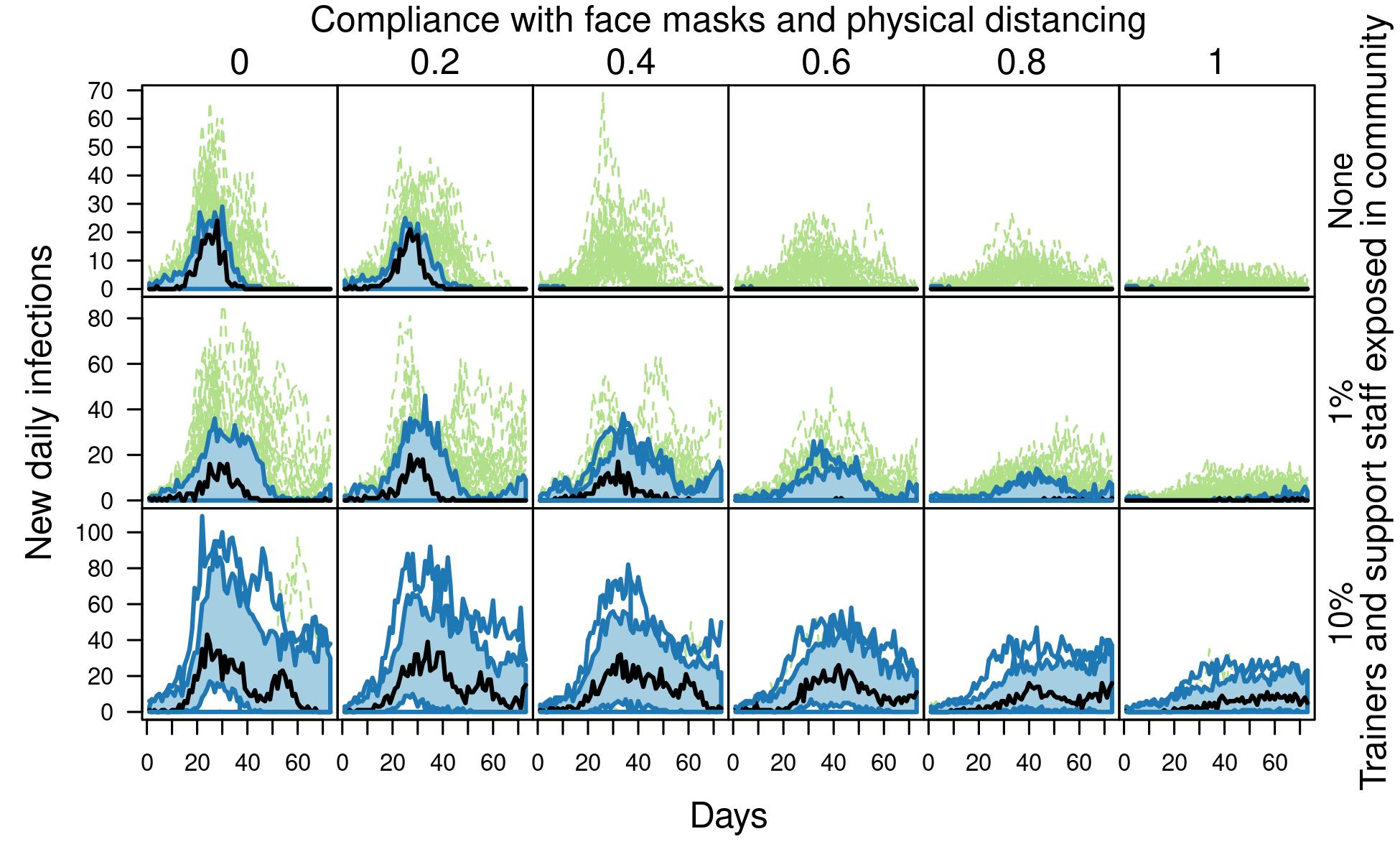

Supplement: S3 Fig — From left to right, columns show increases in the proportion of time that individuals comply with face masks and physical distancing. From top to bottom, rows show increases from 0 to 0.01 to 0.10 of the probability that trainers and support staff were exposed to the virus in the community over the course of the 70-day training period. Each panel shows a functional boxplot of the daily incidence of new infections across 1,000 replicate simulations, showing the median estimate (black line), 50% central region (25–75%)(blue area), 1.5 times the central region (blue lines), and outliers defined as curves outside of the 1.5 times the central region (dashed green lines). (TIF) [file pcbi.1010489.s003.tif]

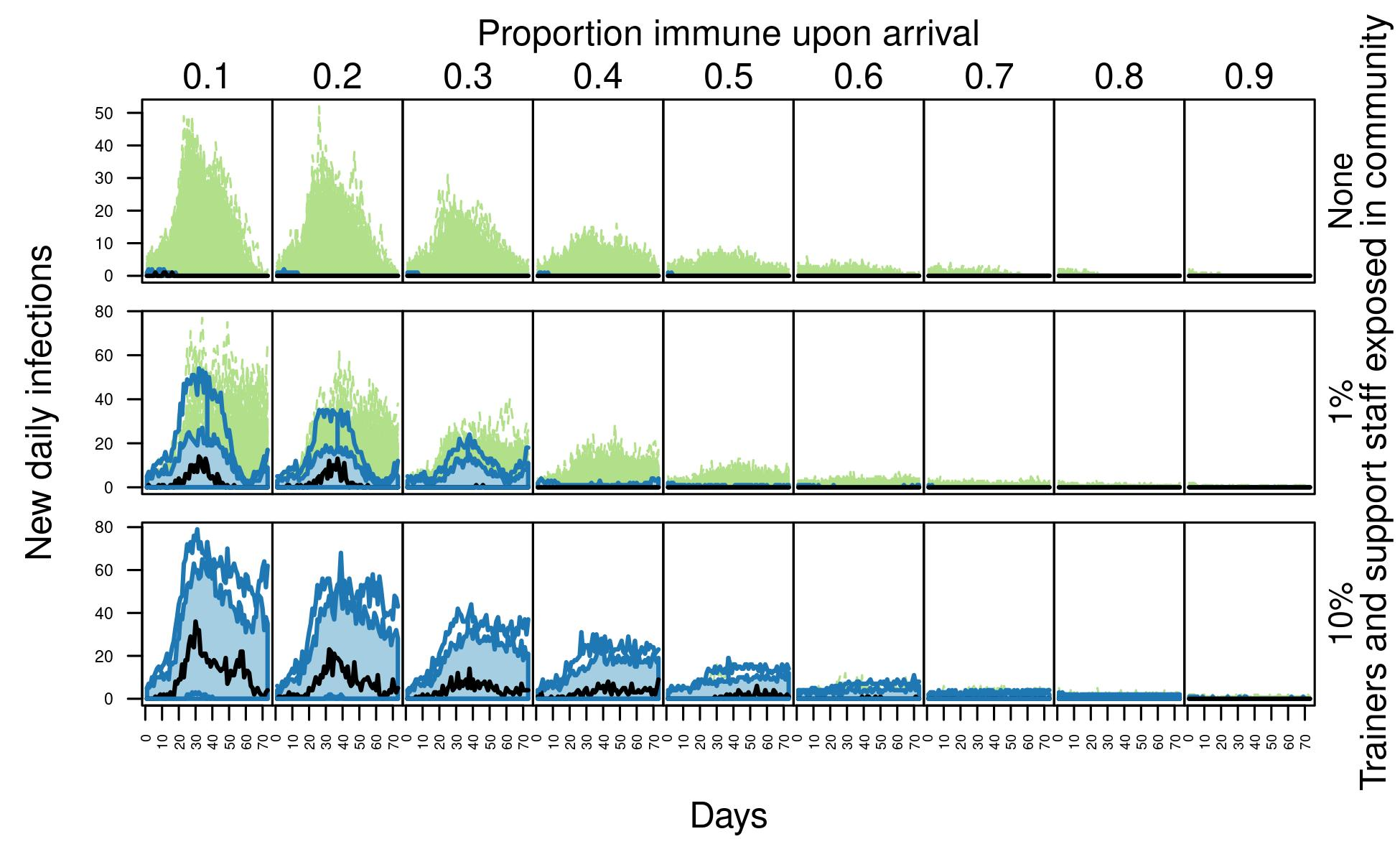

Supplement: S4 Fig — From left to right, columns show increases in the proportion immune upon arrival. From top to bottom, rows show increases from 0 to 0.01 to 0.10 of the probability that trainers and support staff were exposed to the virus in the community over the course of the 70-day training period. Each panel shows a functional boxplot of the daily incidence of new infections across 1,000 replicate simulations, showing the median estimate (black line), 50% central region (25–75%)(blue area), 1.5 times the central region (blue lines), and outliers defined as curves outside of the 1.5 times the central region (dashed green lines). (TIF) [file pcbi.1010489.s004.tif]

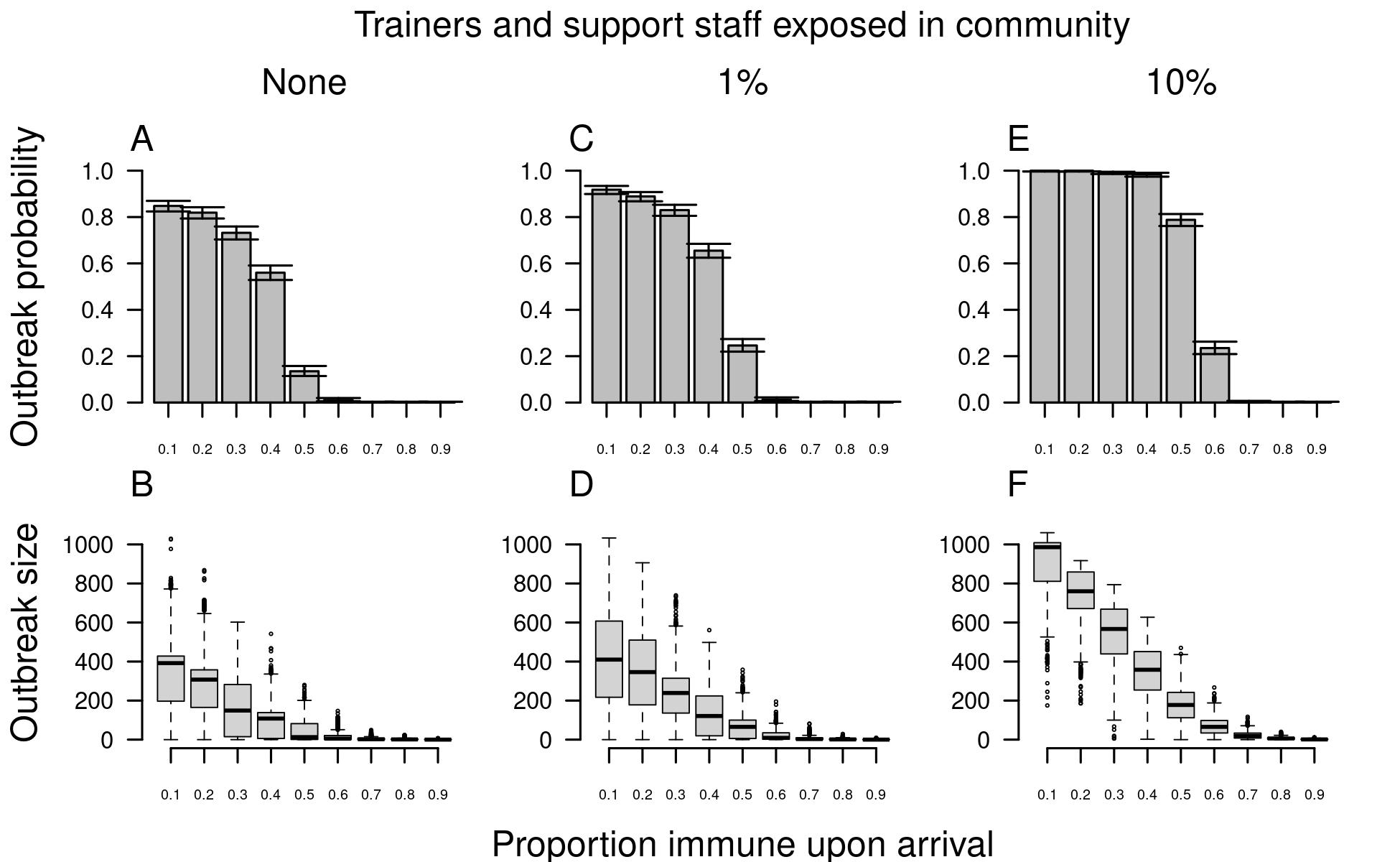

Supplement: S5 Fig — From left to right, columns show increases from 0 to 0.01 to 0.10 of the probability that trainers and support staff were exposed to the virus in the community over the course of the 70-day training period. Error bars for outbreak probability indicate 95% Pearson-Clopper confidence intervals. (TIF) [file pcbi.1010489.s005.tif]

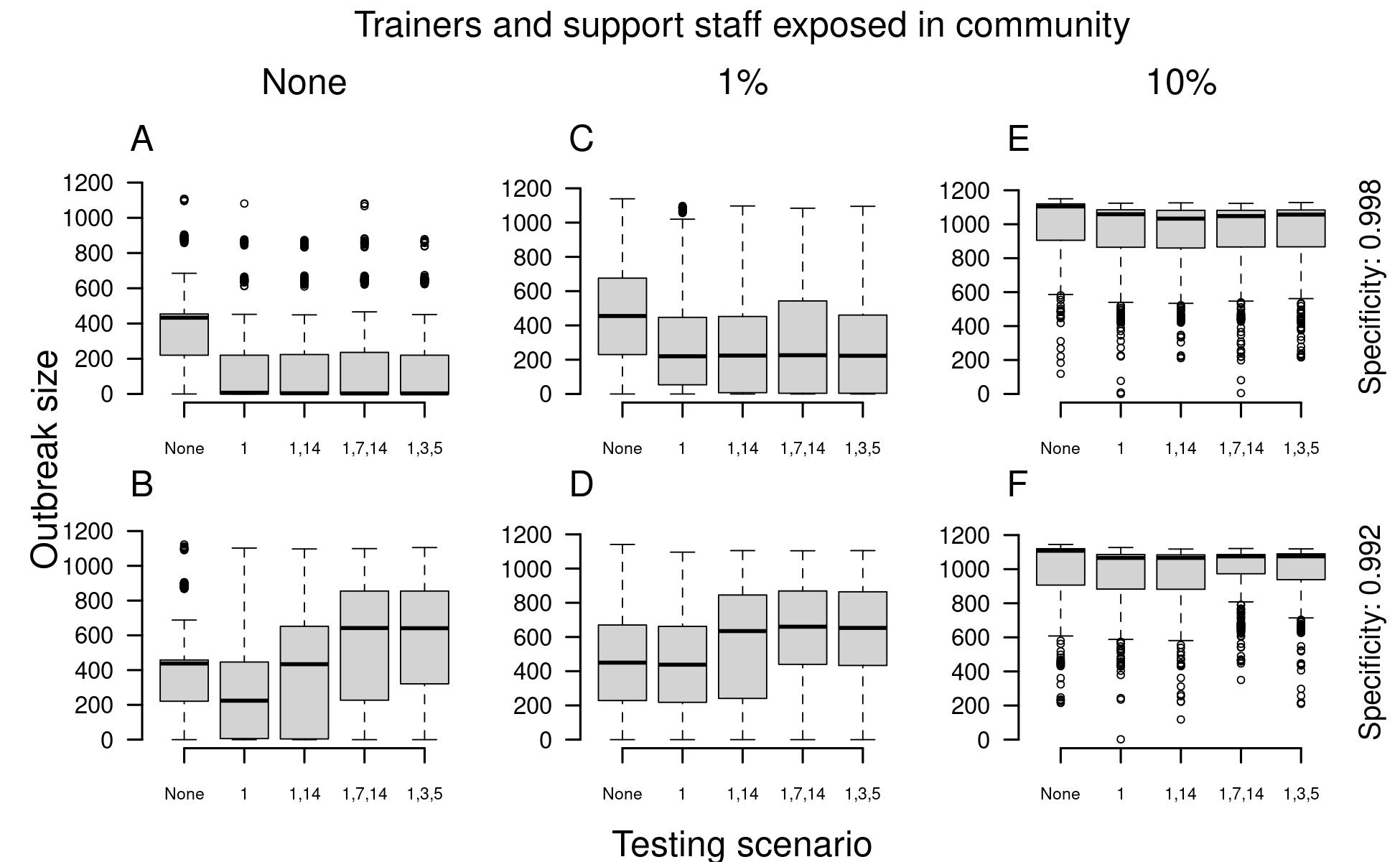

Supplement: S6 Fig — Testing scenarios are labeled according to the day on which a test was administered to trainees following their arrival. From left to right, columns show increases from 0 to 0.01 to 0.10 of the probability that trainers and support staff were exposed to the virus in the community over the course of the 70-day training period. Error bars for outbreak probability indicate 95% Pearson-Clopper confidence intervals. (TIF) [file pcbi.1010489.s006.tif]

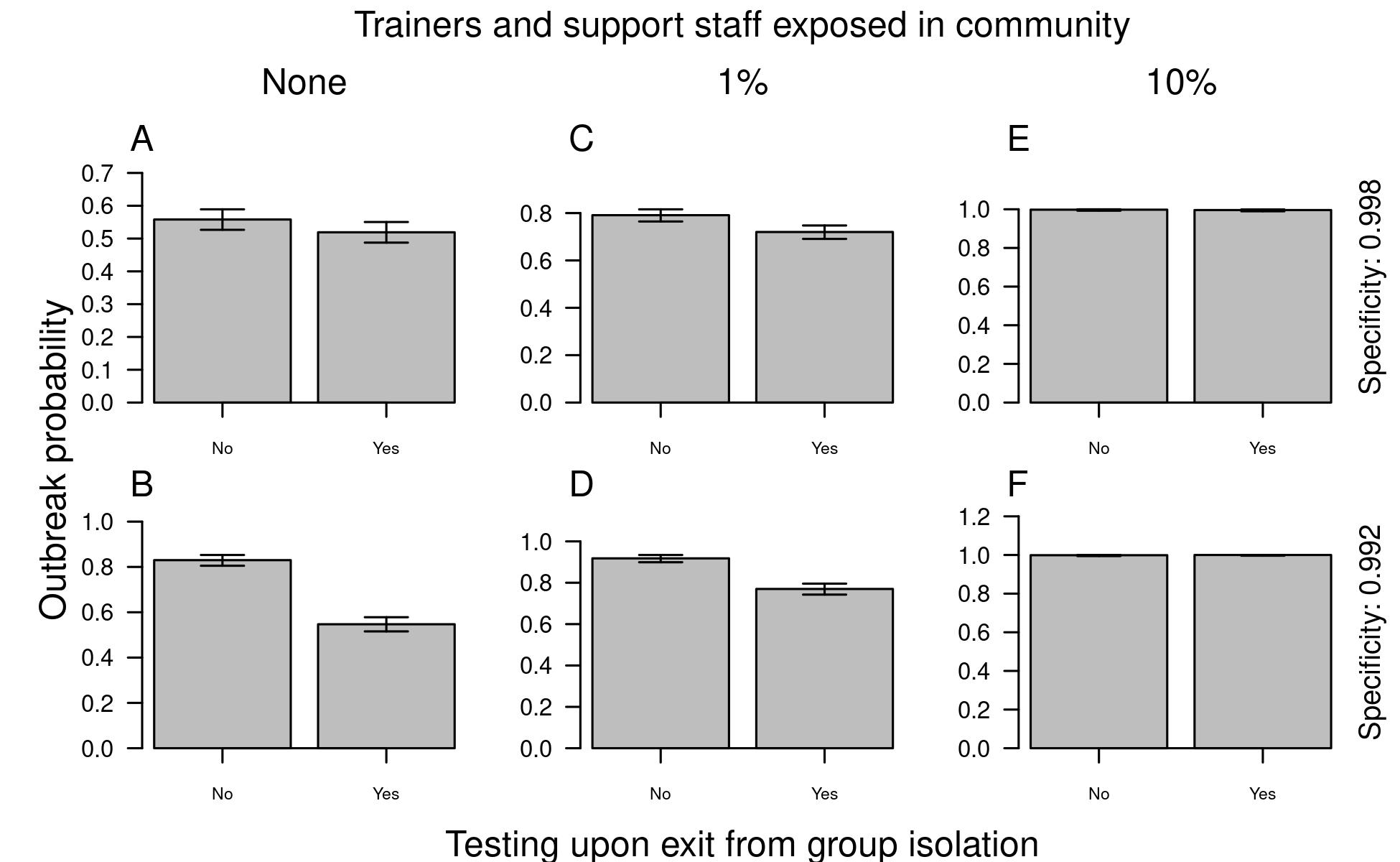

Supplement: S7 Fig — Rows show results for different values of test specificity. From left to right, columns show increases from 0 to 0.01 to 0.10 of the probability that trainers and support staff were exposed to the virus in the community over the course of the 70-day training period. Error bars indicate 95% Pearson-Clopper confidence intervals. (TIF) [file pcbi.1010489.s007.tif]

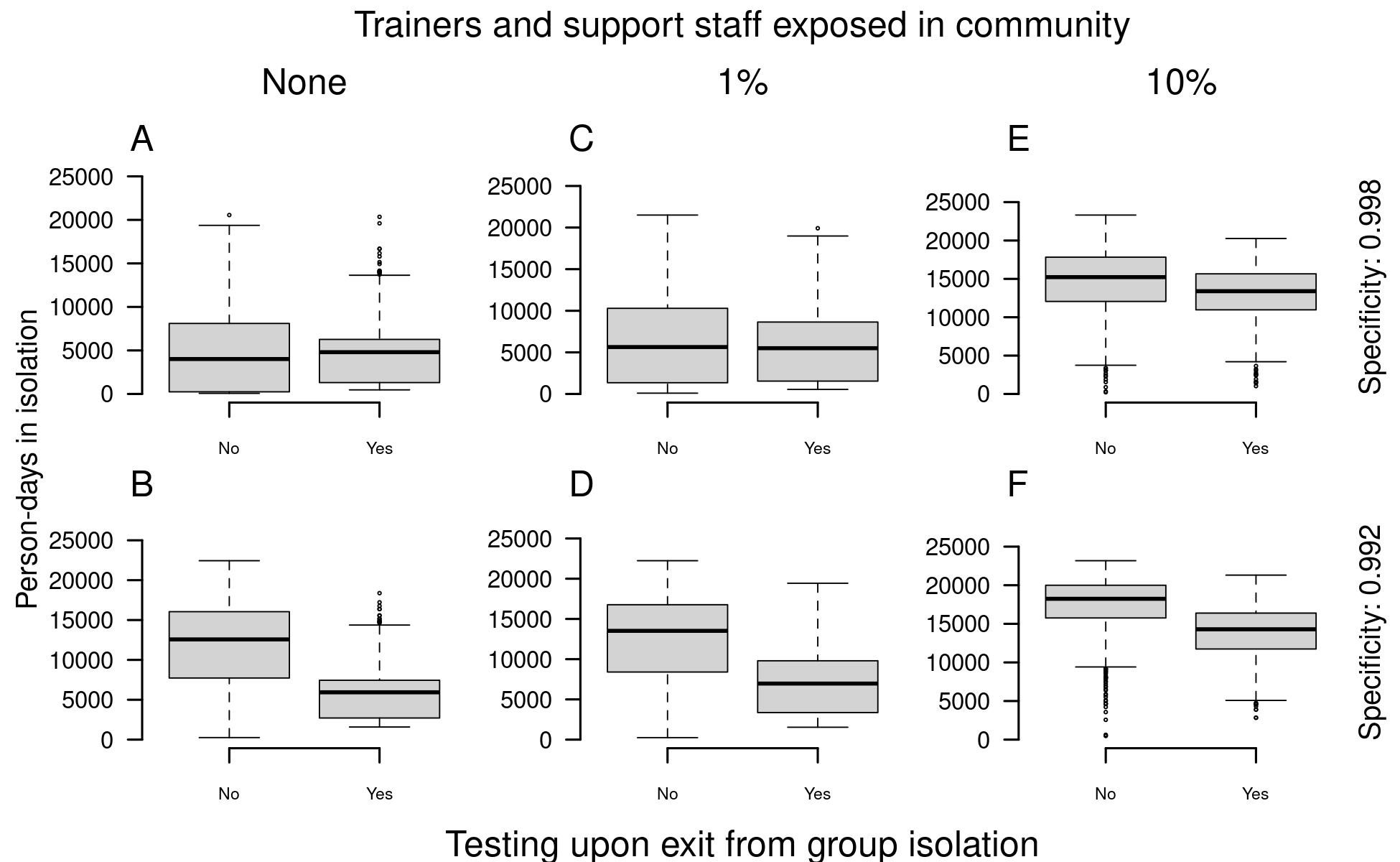

Supplement: S8 Fig — Rows show results for different values of test specificity. From left to right, columns show increases from 0 to 0.01 to 0.10 of the probability that trainers and support staff were exposed to the virus in the community over the course of the 70-day training period. (TIF) [file pcbi.1010489.s008.tif]

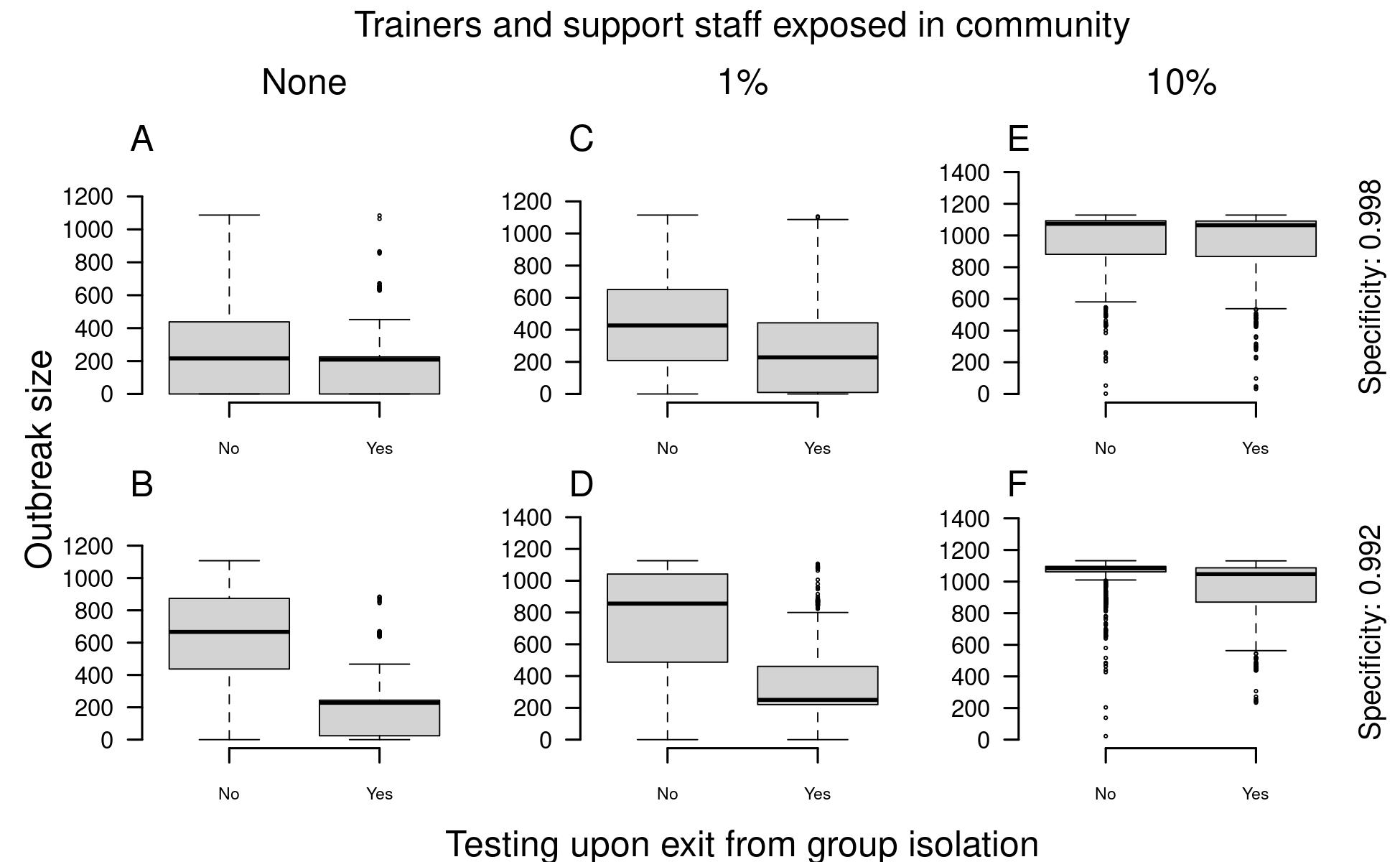

Supplement: S9 Fig — Rows show results for different values of test specificity. From left to right, columns show increases from 0 to 0.01 to 0.10 of the probability that trainers and support staff were exposed to the virus in the community over the course of the 70-day training period. (TIF) [file pcbi.1010489.s009.tif]

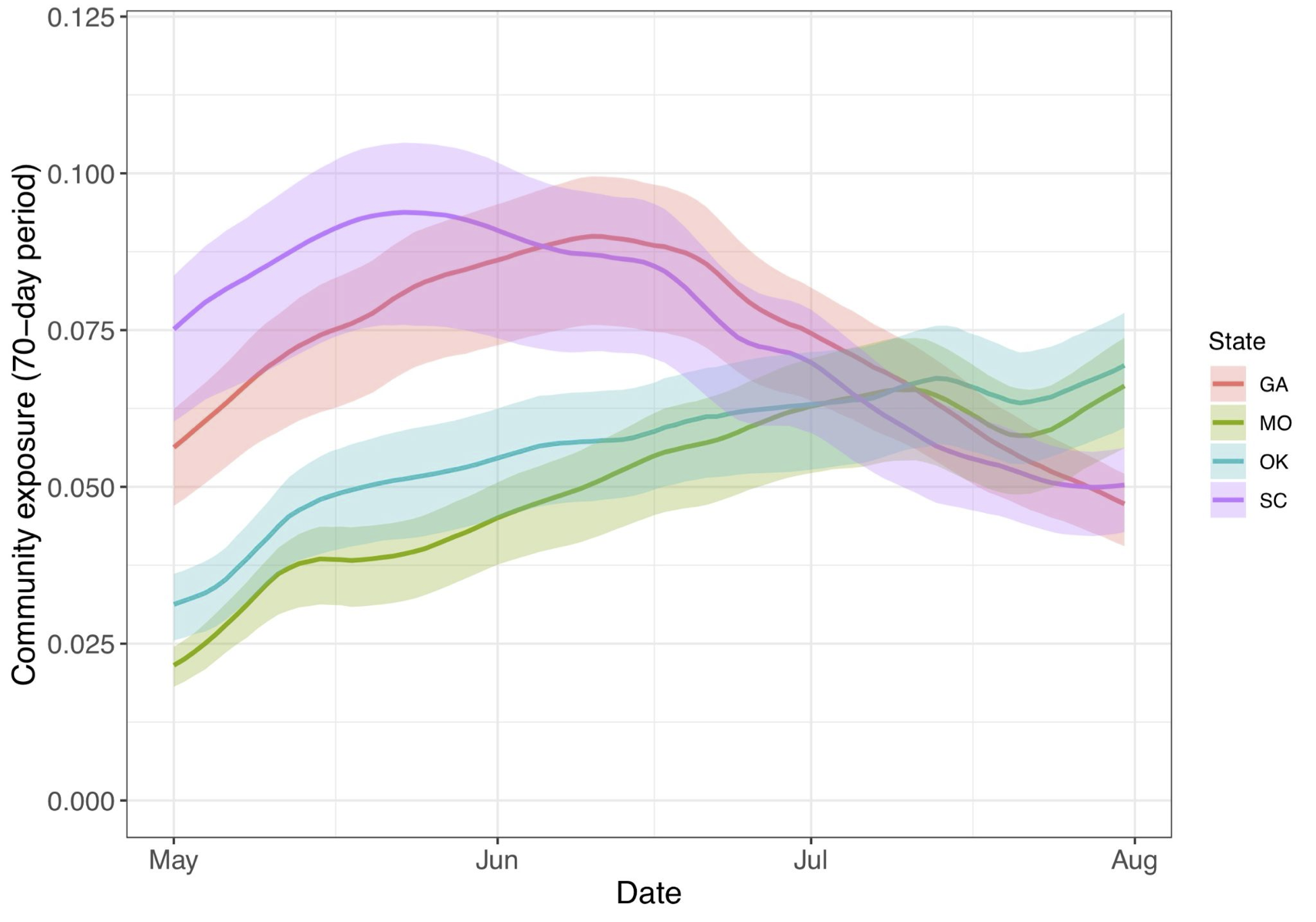

Supplement: S10 Fig — This measure of exposure is defined as infection attack rate over a 70-day period commencing on the date indicated on the x-axis in 2020, as estimated by Pei et al. (24). Solid lines show medians, and bands show 95% credible intervals. (TIFF) [file pcbi.1010489.s010.tiff]

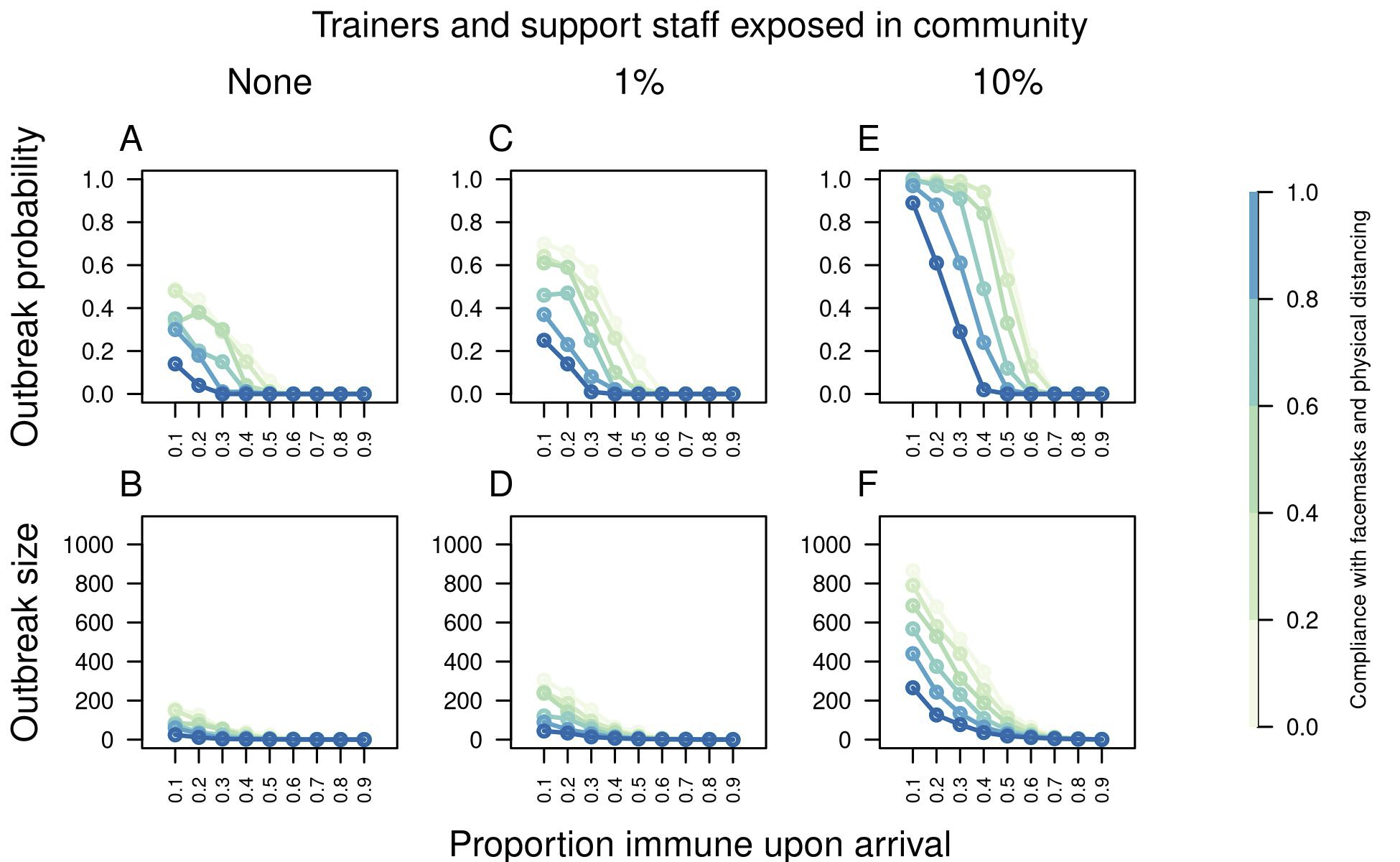

Supplement: S11 Fig — From left to right, columns show increases from 0 to 0.01 to 0.10 of the probability that trainers and support staff were exposed to the virus in the community over the course of the 70-day training period. Each point reflects a proportion or median across 1,000 replicate simulations. (TIFF) [file pcbi.1010489.s011.tiff]
