# Supplementary material for: Prioritizing interventions for preventing COVID-19 outbreaks in military basic training
Source: PLoS Comput Biol. 2022 Oct 7;18(10):e1010489. doi: 10.1371/journal.pcbi.1010489 (PMC9581358; doi:10.1371/journal.pcbi.1010489)
Supplement: Attachment — Submitted filename: Round 2 - PNAS Reviews & Response.pdf [file pcbi.1010489.s012.pdf]

November 10, 2021

Title: "Prioritizing interventions for preventing COVID-19 outbreaks in military basic training"
Tracking #: 2021-04941R
Authors: España et al.

Dear Dr. Perkins,

Thank you for submitting your manuscript "Prioritizing interventions for preventing COVID-19 outbreaks in military basic training" for possible publication in PNAS. We sincerely apologize for the delay in rendering a decision on your manuscript. After an initial evaluation of its general suitability for PNAS, your manuscript was assigned to an editor with expertise in the subject area of your work, who then examined the manuscript, identified 2 expert reviewers in the field, and solicited their assessment of your work. These reviews, which appear below, were then considered by the editor, who determined that the problems pointed out by the reviewers were of sufficient concern to merit rejection of the manuscript. Our goal at PNAS, to publish novel, transformational research rapidly, prevents us from undertaking multiple rounds of revision and re-review after receiving negative reviews. Accordingly, resubmission of the article will not be permitted.

Although we expect that this outcome is disappointing, we hope that the rationale for our decision process is clear and that you found the comments of the reviewers helpful. We thank you for choosing PNAS as a potential venue for publishing your work and we hope that you'll continue to consider PNAS for future submissions.

Yours,
May R. Berenbaum
Editor-in-Chief

****

Editor's Remarks to Author:

I recognize that your revision has nicely taken care of the technical issues, as emphasized by the first reviewer, and the new reviewer. However, the new reviewer raises similar concerns to the previous reviewer who was unavailable. Given this, at this stage I must recommend that a more specialized outlet would be a much better fit; the manuscript unfortunately is not competitive for the limited space in PNAS.

Reviewer Comments:

Reviewer #2:

Suitable Quality?: Yes
Sufficient General Interest?: Yes
Conclusions Justified?: Yes
Clearly Written?: Yes
Procedures Described?: Yes
Supplemental Material Warranted?: Yes

Comments:

Comment 2.1:
The authors have done a very good job resolving my concerns with the paper, both in describing the technical details and in framing its broader interest and applicability.

Response 2.1:
We thank the reviewer for taking the time to review a revised version of our manuscript and are pleased to know that they had a positive assessment of those revisions. We have done additional work to address the minor critiques described below.

I have only a few minor comments.

* CHOICE OF R0 value: baseline, priors, etc.

Comment 2.2:
I completely agree that the observed outbreaks at FB and FLW are not representative/random samples of all army bases, but I'm wondering at the specific rationale for choosing the 0.1% quantile of the posterior distribution based (which seems rather extreme). According to https://www.militaryonesource.mil/military-life-cycle/new-to-the-military/military-career/army-basic-training-and-officer-candidate-school-what-to-expect/ the US Army only has 5 basic training locations [Fort Benning, GA; Fort Jackson, SC; Fort Leonard Wood, MO; Fort Sill, OK; Fort Knox, KY] - from that point of view, FB and FLW would only represent the top 40% of bases in terms of outbreak risk. One could say that the hypothetical sampling universe is larger (all US military bases that do any kind of training?) but I'm still struggling with the choice of 0.1%.

Response 2.2:
Ideally, we would use complete data from all U.S. Army training posts over the duration of the pandemic to inform our estimate of R_0. Unfortunately, such data have not been released, so we are limited to informing our model with data from two large outbreaks that garnered media attention. While these are not the only outbreaks that have occurred, co-authors with knowledge of more comprehensive data suggest that these outbreaks were larger than what is seen most of the time. As such, if we were to focus our analyses on the point estimate of R_0 from our calibration, it would considerably overrepresent the extent of transmission in this setting. Whether it is best to choose the 0.1% quantile or another one is impossible to know without additional data. That said, we do appreciate the reviewer's point and added the following sentence to the Methods

on page 7 to more clearly state our rationale for this choice: *"We chose the 0.1% quantile for R_0 because the outbreaks at FB and FLW were unusually large and because values of R_0 within this range resulted in model behavior with greater sensitivity to changes in other model parameters than higher values of R_0 did."*

Comment 2.3:
Similarly, choosing to use a prior U(2,20) feels much weaker than necessary. Admittedly we know much less about R0 in congregate settings, but we do have some data (refs 40-47 in the manuscript). Just based on background knowledge I would have said that R0<3 and R0>15 were both very unlikely - and uniform distributions are rarely a very good model of the state of our knowledge (i.e., this prior is saying that R0=1.9 or R0=20.1 are completely impossible but that the prior probability of R0=2.1, 6, or 19.9 are identical...). I would consider a log-normal prior based on the moments of the point estimates reported in refs 40-47.

Response 2.3:
To address the reviewer's concern, we informed the prior for R_0 using a normal distribution, with a mean and standard deviation obtained from a combined estimate of three different values found in the literature related to R_0 estimates in congregate settings. The mean R_0 of this prior distribution was defined as 9.6 (95% CI: 6.9 - 12.4). References used to inform this are now cited in the text and listed below for your convenience.

https://www.cdc.gov/mmwr/volumes/69/wr/mm6931e1.htm
https://elifesciences.org/articles/58699
https://www.sciencedirect.com/science/article/pii/S1047279720303471

Comment 2.4:
The authors comment (l. 639) that R0 had "extremely wide ranges of uncertainty in our model calibration" - this could be a symptom of failing to use existing information about R0 in congregate settings.

Response 2.4:
After updating our prior distribution consistent with the reviewer's suggestions, we found similarly wide ranges of uncertainty about R_0 following our model calibration, with resulting upper boundary values of $R_0 > 11$.

Comment 2.5:
Bottom line, I feel that the authors might have done a little bit of informal or off-the-books Bayesianism, choosing values and ranges that *they knew to be reasonable* from among the range of possibilities...

Response 2.5:
The only parameter where some subjectivity was involved in selecting a baseline value was R_0. As explained in Response 2.2, this was necessary given the paucity of data to inform this parameter value. The reviewer's suggestion to better inform this parameter

with a more informed prior did not make much of a difference. Ultimately, we had to select a value based on a combination of acknowledging the rarity of the large outbreaks used to inform the model and our desire to use a value of R_0 that would allow our sensitivity analysis to reveal the sensitivity of the model's behavior to changes in a wide variety of parameters. After all, the primary goal of this study was not to make specific quantitative predictions but to aid decisionmakers by developing their intuition about the effects that alternative policies could have on outbreak dynamics in this setting. Besides R_0, all other parameters were well informed by existing literature or expert opinion by individuals knowledgeable about this setting. As shown in the sensitivity analysis, higher values of R_0 make outbreak dynamics less sensitive to changes in other model parameters, meaning that a higher baseline value of R_0 would have masked those effects and impaired our presentation of results about them.

* FIGURES

Comment 2.6:
I appreciate that the authors followed up on my suggestions about functional boxplots and binomial CIs. It's not necessary, but I wonder if violin plots (possibly with medians/IQRs superimposed) would be more informative for the outbreak size plots (e.g. Figure 5B, 6B, 7B)?

Response 2.6:
We prefer the reviewer's previous suggestion to use functional box plots and have decided to retain that choice in this version of the manuscript.

* TESTING DELAYS

Comment 2.7:
The value of one day for a testing delay seems reasonable given the context. It's outside of the current scope of the analysis, but I would be very interested in knowing the sensitivity of the results to changes in testing delay (between, say, 0.5 [which would require changing the time step and hence be a lot of work) and 4 days (an extreme value for a well-organized military base, but conceivable under some circumstances) - and especially the importance of a 1- vs 2-day delay, which might have very practical implications for allocation of resource to test processing facilities.

* REFERENCES

Comment 2.8:
There are a few places (ref 29, ref 51, ref 53) where the references look a little bit garbled.

Response 2.8:
We have updated these references so that they are now more complete and presentable.

* ARCHIVING

Comment 2.9:
I would encourage the authors to deposit their code in an archive on Zenodo.org (this is easily done from a GitHub repository).

Response 2.9:
We appreciate this suggestion and plan to do so after the final version of the manuscript has been accepted.

Reviewer #3:

Suitable Quality?: No
Sufficient General Interest?: No
Conclusions Justified?: Yes
Clearly Written?: No
Procedures Described?: Yes
Supplemental Material Warranted?: Yes

Comments:
This new reviewer (expertise in network science x behavioral science x simulations) has read the revised article and seen several unsolved issues in the revised manuscript.

Comment 3.1:
First, this reviewer agrees with the Original Reviewer #2.1 ("I'm not sure it's sufficiently novel/of broad interest to warrant publication in PNAS") and has a concern - only a few readers (who are interested in the US military basic training) will read the paper if the paper stays at the current format and style. Although schools, summer camps, concerts, cruise ships, etc. share some "gathering" features, the results of the simulations and policy implications based on the simulations will be highly contextual. I have not seen that the army setting uniquely provides a rare setting for advancing science broadly (while Diamond Princess was a great setting to calculate many parameters of COVID-19 in early 2020 [but yes, the broad audience have no longer been interested in cruise ships as of Nov 2021]).

Response 3.1:
We feel that we have already provided a thorough response to this critique in Response 2.1 following the first round of reviewer comments. Importantly, as indicated above, Reviewer 2 (who originally raised these concerns) was satisfied with our response and changed their assessment to “Sufficient General Interest?: Yes.” We feel that this new reviewer has provided no additional substantive critique on this issue that we have not already responded to.

Comment 3.2:
Although authors have investigated four intervention techniques (reducing gatherings, arrival testing, behavioral change [facial coverings and distancing], and vaccination) in simulations with the military setting, these four techniques themselves are not novel (but examined elsewhere already). It is easy to imagine that the effect of arrival testing or vaccination will depend on the accuracy and/or the effectiveness of these measures (even without simulations). The revised manuscript has not yet provided a novel solution to the pandemic or a solution to the next wave of SARS-CoV-2 or future unknown pathogens. Although the authors suggested Kasper et al, 2020 and Letizia et al, 2020 (the two NEJM papers) as well as many papers using the Diamond Princess got broad attention in 2020, this reviewer thinks that what we want to read as of November 2021 has changed. For example, since the author team has Army officers and senior leaders, qualitative or quantitative research regarding how this type of simulation result could affect high-level decision-making in the Army setting may be more interesting, as compared with a simple display of the simulation results with a broad range of parameter values.

Response 3.2:
There are several points to respond to here.

First, we agree with the reviewer that many modeling studies have evaluated the impacts of the interventions we considered, both in isolation and in combination. The novelty of our work is in evaluating the effects of those interventions in the highly specific context of military basic training. We are not aware of any work that has evaluated this set of interventions in this or any comparable setting and, as we show in the manuscript, there are unique aspects of how these interventions work in such a setting. Indeed, that is the main thrust of our manuscript.

Second, we feel that the reviewer's critique that *"The revised manuscript has not yet provided a novel solution to the pandemic or a solution to the next wave of SARS-CoV-2 or future unknown pathogens."* is unreasonable in the scope of what it expects us to address in this manuscript. To us, this is analogous to criticizing a manuscript evaluating a therapy for a certain type of cancer for not proposing a cure for all cancers. It is simply unreasonable. With respect to the context of military basic training, the context of our study has proven quite durable, with waning immunity and new variants leading to the threat (and occurrence) of outbreaks in this setting persisting beyond the timeframe that many expected initially. Despite changes in vaccination and variants over time, the same tools that we evaluate here remain cornerstones of outbreak prevention and control in this setting.

Third, the reviewer's comment that *"how this type of simulation result could affect high-level decision-making in the Army setting may be more interesting"* is ironic given that we proposed to do precisely that in a companion piece to be published alongside our manuscript. Please see below for text included in our original cover letter proposing this idea to the editor.

“Should this manuscript be accepted, we also hope that you would consider publishing a companion piece, possibly as a Commentary, to provide an opportunity for military commanders to offer context on the significance and real-world benefits of this research. We believe a Commentary prepared by a senior military commander (Brigadier General Beagle) and our team would be of great interest to your audience and would bridge the gap from academic exercise to practical decision making by leaders charged with preserving the health and welfare of the military while at the same time preserving the training mission and maintaining our national defense capability. This Commentary would communicate the practical application of infectious disease modeling to military decision making in the context of a military population that is integrated into the surrounding civilian community.”

Comment 3.3:
By the way, from the technical perspective, I think the authors have revised the manuscript nicely this time (although this reviewer did not read the original submission very carefully, though).

Response 3.3:
We are happy to hear that the reviewer is satisfied with the technical aspects of our manuscript.
