# Supplementary material for: Prioritizing interventions for preventing COVID-19 outbreaks in military basic training
Source: PLoS Comput Biol. 2022 Oct 7;18(10):e1010489. doi: 10.1371/journal.pcbi.1010489 (PMC9581358; doi:10.1371/journal.pcbi.1010489)
Supplement: Attachment — Submitted filename: Response to Reviews_BasicTrainingModel.pdf [file pcbi.1010489.s014.pdf]

REVIEWER 1

Comment 1.1
This is a clearly, well-written manuscript. The methodology is appropriate and described in sufficient detail. This reviewer mainly has some comments on the figures, and a few minor ones on the main text.

Response 1.1
Thank you for the positive appraisal of our work.

Introduction

Comment 1.2
Even though you do mention it in the methods, I think it would be helpful to the reader to specify in the introduction the length of training (70 days), as well as the type of test used (PCR).

Response 1.2
We have now added these two points in the third paragraph of the Introduction.

Methods

Comment 1.3
On page 22, in the *Pre-arrival vaccination* section, you state "COVID-19 vaccines appear highly effective and are anticipated to become increasingly available over the course of 2021..." I would suggest rephrasing that sentence to reflect what happened in 2022.

Response 1.3
We revised this section as follows to make it appropriate for a more contemporary readership.

"Immunity among trainees, trainers, and support staff varies naturally depending on the history of the epidemic in communities that these individuals come from and the time in the epidemic when they arrive. Similarly, vaccination coverage varies across communities, as well. For both forms of immunity, waning can compound variability in protection against infection at different points of time in different groups of people. To cover this wide range of possible scenarios, we varied the proportion immune upon arrival from 0 to 90% in increments of 10%."

Comment 1.4
It would be interesting to explore the effect of combined interventions, eg. increased immunity as well as masking compliance. Considering the current uptake of covid vaccines, it would be beneficial to examine the effects of such interventions on immunity levels greater than the baseline use in the model.

Response 1.4

In response to this comment, we performed an additional set of simulations described in the new passage below, which we added to the Results in the “Pre-arrival vaccination” subsection of the “Impacts of interventions” section. The new supplemental figure that this text refers to is also included below.

“In general, for high levels of compliance with facemasks, the levels of immunity required for the probability of an outbreak to be zero was lower (Fig. S11). For instance, assuming high levels of introductions and 100% compliance with facemasks, previous immunity would need to be 40% to result in the probability of an outbreak to be zero. In contrast, at no compliance with facemasks, previous immunity would need to be 70% to achieve the same benefits (Fig. S11E).”

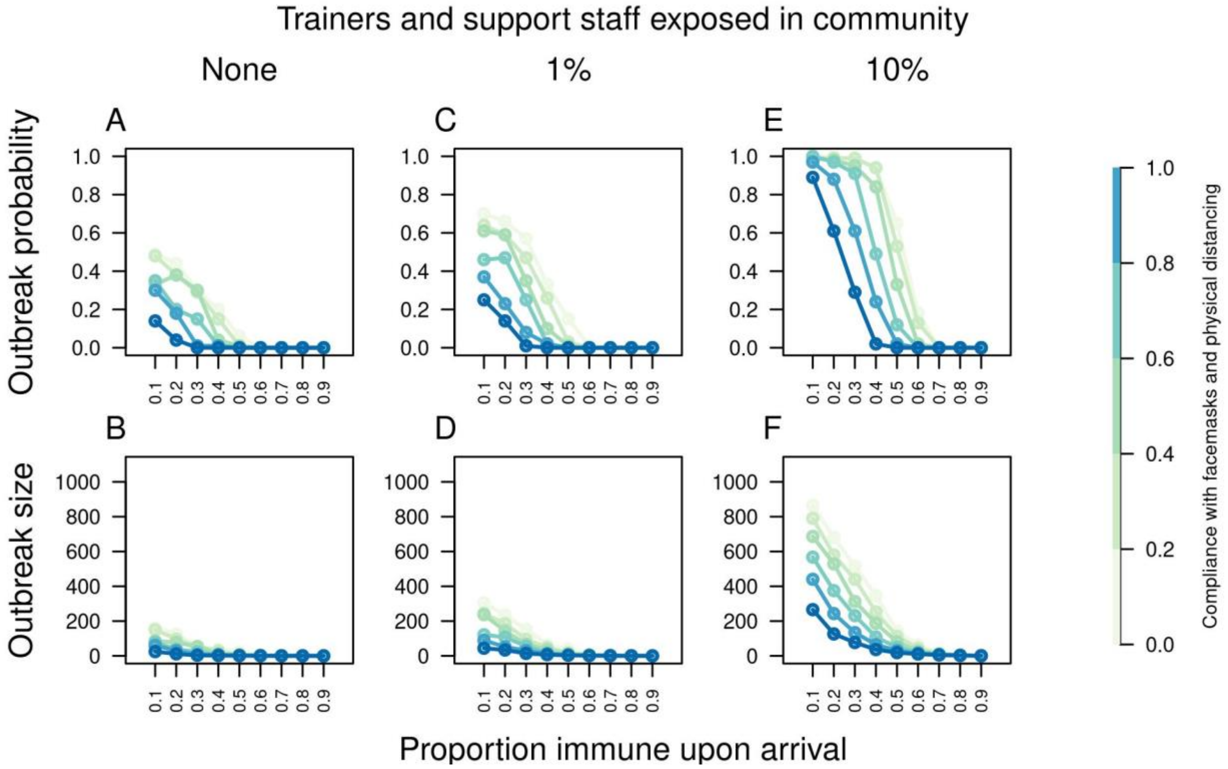

Figure S11. Outbreak probability (top) and size (bottom) in basic training as a function of the proportion immune upon arrival (x-axis) and compliance with facemasks and physical distancing (colors). *From left to right, columns show increases from 0 to 0.01 to 0.10 of the probability that trainers and support staff were exposed to the virus in the community over the course of the 70-day training period. Each point reflects a proportion or median across 100 replicate simulations.*

Discussion

Comment 1.5
you mention that your results are applicable to determining customized strategies for preventing COVID-19 outbreaks. This seems like a missed opportunities to talk broadly about emerging respiratory infectious diseases and how this work can be implemented in such cases, where the level of immunity is low. I would recommend adding one or two sentences commenting on that.

Response 1.5
In response to this comment, we added the following sentence to the very end of the Discussion.

"In the event of other emerging respiratory viruses in the future, our model could serve as a starting point for exploring the possible impacts of such a virus and how best to control it to maintain basic training operations."

Figures

Comment 1.6
On Figure 2, both A and B, I cannot see the dashed green lines (at least the cases that fall above 97.5%)

Response 1.6
The caption was incorrect. The blue lines do not represent the 2.5-97.5% interval. Rather, those lines represent the bounds of the 1.5 times the 50% central region. Outliers are detected as curves outside of the fences defined by the 1.5x central region. These are standard settings for the functional boxplots we used. We have updated the figure captions to clarify this, as shown below.

"The functional boxplot shows the median estimate (black line), 50% central region (25-75%) (blue band), 1.5 times the central region (blue lines), and outliers defined as lines outside of 1.5 times the central region (dashed green lines)."

Comment 1.7
On Figure 3B, I cannot see the black line for the median.

Response 1.7
The median curve for these figures is 0, which is why it cannot be seen.

Comment 1.8
On Figure 4B, I cannot see the black line for the median.

Response 1.8
The median curve for these figures is 0, which is why it cannot be seen.

Comment 1.9
On Figures 4B, and S3 and S4, I am having trouble understanding the green dashed lines. These lines are the outliers—cases above the 97.5%. With 1000 replicates it should be 25 lines. In many of these panels (such as the topleft panel of Figure S4) it looks like it's way more than 25 lines.

Response 1.9
As mentioned in Response 1.6, there was an error in the captions explaining the functional boxplots. In response to these comments, we have edited the captions as follows to address the issue noted by the reviewer. We note that, under this definition of what constitutes an outlier, there is no clear number of outlier lines that would be expected in a plot such as this.

"The functional boxplot shows the median estimate (black line), 50% central region (25-75%) (blue band), 1.5 times the central region (blue lines), and outliers defined as lines outside of 1.5 times the central region (dashed green lines)."

REVIEWER 2

Comment 2.1
The authors model COVID outbreak in a military basic training setting. They estimate a parameter space based on the outbreaks at Forth Benning and Fort Leonard Wood, and with the findings from several published studies (barring the relative infectiousness of asymptomatics). They construct a baseline simulation and compare its results to simulations which vary the parameter space to test the effects of 1) reducing introductions by trainers and support staff, 2) increasing testing of trainees, 3) increasing compliance with wearing face masks and physical distancing, and 4) increasing immunity among trainees through pre-arrival vaccination. The results of the simulations are very informative but not very surprising. However, I think this paper remains a quality addition to the growing library of COVID related research. I have several minor concerns regarding the methodology, and a few concerns about knowledge translation.

Response 2.1
We thank the reviewer for taking the time to review our manuscript and provide constructive comments.

Comment 2.2
In the last paragraph of your introduction, consider changing "That informed the model's assumptions ..." to an alternative such as "The calibrations informed the model's assumptions ..." to minimize ambiguity.

Response 2.2
We have revised the manuscript in accordance with the reviewer's suggested wording.

Comment 2.3
In the last paragraph of the introduction you first mention the effect of introductions by trainers and support staff, however I could not find a passage where you outline what introductions entail. Introductions vary between cultures, and I imagine between civilian and military settings. A followup question is whether there are intermediate levels of introduction.

Response 2.3
We elaborated on what we mean by introductions by adding the following additional wording in the passage that the reviewer referred to: *"introductions of the virus into the basic training setting."*

Comment 2.4
I thought figure 1 was a great pictorial representation of your model, but I would suggest a possible improvement. The transitions and contact column could be represented as a state-diagram/flowchart. This alternative representation would be appealing/recognizable to biologists (e.g. life history cycles) and computer scientists, alike, and might improve knowledge translation.

Response 2.4
We appreciate this suggestion, but we were unable to modify the figure in this manner while still maintaining its contents and font size for the text it contains. We hope that the reviewer and readers will be satisfied with the figure in its current form.

Comment 2.5
In your results, first paragraph under "model calibration", you first reference figure 2. There is no mention within the paragraph or the figure caption for why the outliers (dashed green lines) are not visible – are the outliers superimposed or negligible?

Response 2.5
Based on how we defined the blue band and lines (which we clarified above in Response 1.9), it just so happens that there were no outliers in Figure 2 according to this definition. As such, we have now removed the mention of outliers and dashed green lines in the caption for Figure 2.

Comment 2.6
In your results, second paragraph under "model behaviour under baseline scenario" you say, "According to this definition, 71% of simulations resulted in an outbreak ...", and shortly after "... the probability of an outbreak increased to 0.95 ...". I would suggest using one representation of probability, rather than two, to minimize ambiguity.

Response 2.6
In both cases, we are referring to the same probability. In the first passage that the reviewer refers to, we are simply referring to the definition of outbreak that we provided in the previous sentence: *"we defined an outbreak as a simulation in which 100 or more infections occurred over the training period."* We felt that it was important to connect

back to this definition in the sentence that begins *"According to this definition"* but that using the term *"probability of an outbreak"* would be sufficient thereafter. While we can appreciate the reviewer's suggestion to simplify this, we feel that it is important to be clear about how we define an outbreak given that that definition is not obvious based on the word itself. To try to help address the reviewer's concern, we added *"(which we refer to as an outbreak hereafter)"* immediately after *"According to this definition"* to draw further attention to the fact that we use this definition of outbreak consistently throughout the manuscript.

Comment 2.7
In your results, figure 8, the y-axis labels should either be written out entirely or explained in the figure caption.

Response 2.7
In response to this comment, we have now written out the y-axis labels in this figure as recommended by the reviewer.

Comment 2.8
In your methods, first paragraph under "model description" you say, "... based on hypothetical assumptions provided by an author (PTS) familiar with operations in this setting". Is it necessary to cite the author specifically because the information is not available publicly? Otherwise, it seems redundant to say that your research is contingent on its authors' knowledge, in which case I would suggest an alternative like "based on informed hypothetical assumptions from the operations in this setting.".

Response 2.8
We have revised the manuscript in accordance with the reviewer's suggested wording.

Comment 2.9
In your methods, first paragraph under "model description", you mention that you use the R programming language. You should include the version of R you used and any relevant packages. As well, as cite the literature associated with those packages (if not base R). You also say a single realization of the model takes around a second to execute on a personal computer, but you do not specify whether this is on single or multiple threads, the operating system, or the CPU you used, all which have a bearing on speed, especially while running multiple instances. I assume the amount of RAM required is negligible.

Response 2.10
We have incorporated these suggestions in the revised paragraph below, which is now the second paragraph of the Methods section. We believe that this level of detail in our description of computing time and resources is sufficient to give readers an adequate understanding of the requirements to run our model. In short, it is not an especially computationally intensive model, and most readers should be able to run it relatively easily and quickly.

“The model was implemented in the R programming language version 4.0 (51) using the packages scam (52) and igraph (53). A single realization of the model takes around a second to execute on a personal computer using Linux (Fedora 34) on a single thread. The simulation analyses were performed using the supercomputing infrastructure of the Center for Research Computing at Notre Dame (https://crc.nd.edu). Different realizations of the model were simulated in parallel, but each simulation was performed on a single-thread computing node. All code used in this analysis is available at https://github.com/confunguido/prioritizing_interventions_basic_training.”

Comment 2.11
In your methods, under “Structuring of agents and their contacts” you say “Trainees arrived over a three-day window and proceeded to one of 20 cocoons of 60 recruits each. After 14 days, five companies of 240 recruits each were formed by pooling together four cocoons.”. You do not mention whether there are any considerations on how recruits are organized into their cocoons, or how cocoons are organized into their companies, in silico or in reality. For example, are trainees/cocoons randomly selected? I would also ask the following hypothetical: if the probability of infection was known for all individuals, how would you distribute them in to cocoons and companies in order to minimize overall transmission?

Response 2.11
We agree that this aspect of the model could be clearer, so in response we added the following sentence.

“Pooling of individuals into cocoons and cocoons into companies was done randomly in the model and not with respect to vaccination status, gender, or any other factor.”

While we have had some discussions with military officials about possible strategies for assembling cocoons and companies in a manner informed by prior infection or vaccination status, we ultimately decided to leave that topic out of this manuscript. It could be an interesting direction for future work though.

Comment 2.12
In your methods, under “Model calibration”, in the third paragraph (following your formulation of the likelihood of p), you say, “The initial set of particles were comprised of draws of p from the estimated distribution combined with draws of R0 from a normal prior distribution ...”. A normal distribution can take-on negative values, whereas the R0 cannot. I am curious as to why you did not choose something like a lognormal distribution? For example lognormal priors have been used to estimate R0 directly (Purkayastha, 2021; https://doi.org/10.1186/s12879-021-06077-9) and indirectly (Mbuvha, 2020; https://doi.org/10.1371/journal.pone.0237126). Some research has also explored using generalized gamma distributions, where a lognormal distribution is a special case.

Response 2.12
We feel that this is a very reasonable suggestion, and it is also in line with a comment by a previous reviewer when our manuscript was previously under consideration at *PNAS*. When responding to that previous comment, we did consider a lognormal distribution for the prior and sought to inform it with R_0 estimates from other institutional settings comparable to that of military basic training. When we did that, we found that the lognormal distribution that resulted was, in our opinion, not wide enough to reflect an appropriate amount of prior uncertainty about this parameter. When we tried a normal distribution, which is what we ended up using, we felt that it resulted in a more appropriate amount of prior uncertainty that did a better job of reflecting the studies whose estimates were used to inform it. Although the reviewer's point about the possibility of R_0 values less than zero is valid, the probability of that occurring given our parameter values is less than 10^{-11}, in which case we feel that it is acceptable to use a normal prior.

Comment 2.13
In your analyses, under "Reducing introductions by trainers and support staff" you do not state your methodology as compared to the "Pre-arrival vaccination" section where you present the same 'one-hand-other-hand' intro, and then later state exactly how you vary your parameters. You also state how you vary your parameters in other nearby sections, just not in "Introductions by trainers and support staff".

Response 2.13
We agree with the reviewer that this should be expanded on. In response to this comment, we added the following sentences immediately after the previous passage that to which the reviewer referred.

"To understand the implications of different rates of introduction by trainers and support staff, we performed simulations under three different levels of introduction (0, 1%, 10%), defined as the proportion of trainers and support staff infected in the community over the course of the 70-day training period. This broad range of variation should cover the full range of possible exposure during this time window, including periods of low and high levels of community transmission."

Comment 2.14
I would like to add to one of your previously addressed comments on isolation before arrival. You say the army does not have control over trainees before arrival and could therefore not rely on a voluntary isolation period of two weeks. Canada has set this very protocol for its recruits, and I would like to think it's due to its efficacy. Yes, you can not expect perfect compliance, but partial compliance must still be effective? I do not know if there is data published on the effect of isolation before arrival for the Canadian military, but it is certainly something worth looking into.

Response 2.14
It is both interesting and encouraging that such an approach has been pursued in Canada. We were not able to locate information about the effect of isolation before

arrival for the Canadian military that could be cited, however. As far as the US context is concerned, we will respect our US military counterparts' views on this given their deep familiarity with the particulars of US military basic training.

Comment 2.15

You do not finish your discussion with a definitive statement on how you think your model will inform decision-making in a military setting. You also state in your previous set of reviewers comments that you have planned a companion piece to express the "real-wold benefits of [your] research". I would suggest that you hint at this future work somewhere in your conclusions.

Response 2.15

In response to this comment, we added the following sentence to the conclusion, which hints at a real decision that actually was informed by our model. We prefer not to state that that action was taken in this manuscript though, given our military counterparts' desire to comment on that separately.

"Likewise, it draws attention to the high priority that should be placed on trainers and support staff for vaccination, which may be a more actionable recommendation based on this work."
