# Supplementary material for: Prioritizing interventions for preventing COVID-19 outbreaks in military basic training
Source: PLoS Comput Biol. 2022 Oct 7;18(10):e1010489. doi: 10.1371/journal.pcbi.1010489 (PMC9581358; doi:10.1371/journal.pcbi.1010489)
Supplement: Attachment — Submitted filename: Espana et al Review.docx [file pcbi.1010489.s013.docx]

This is a clearly, well-written manuscript. The methodology is appropriate and described in sufficient detail. This reviewer mainly has some comments on the figures, and a few minor ones on the main text.

Introduction

Comment 1: Even though you do mention it in the methods, I think it would be helpful to the reader to specify in the introduction the length of training (70 days), as well as the type of test used (PCR).

Methods

Comment 2: On page 22, in the *Pre-arrival vaccination* section, you state “COVID-19 vaccines appear highly effective and are anticipated to become increasingly available over the course of 2021...” I would suggest rephrasing that sentence to reflect what happened in 2022.

Comment 3: It would be interesting to explore the effect of combined interventions, eg. increased immunity as well as masking compliance. Considering the current uptake of covid vaccines, it would be beneficial to examine the effects of such interventions on immunity levels greater than the baseline use in the model.

Discussion

Comment 4: you mention that your results are applicable to determining customized strategies for preventing COVID-19 outbreaks. This seems like a missed opportunities to talk broadly about emerging respiratory infectious diseases and how this work can be implemented in such cases, where the level of immunity is low. I would recommend adding one or two sentences commenting on that.

Figures

Comment 5: On Figure 2, both A and B, I cannot see the dashed green lines (at least the cases that fall above 97.5%)

Comment 6: On Figure 3B, I cannot see the black line for the median.

Comment 7: On Figure 4B, I cannot see the black line for the median.

Comment 8: On Figures 4B, and S3 and S4, I am having trouble understanding the green dashed lines. These lines are the outliers—cases above the 97.5%. With 1000 replicates it should be 25 lines. In many of these panels (such as the topleft panel of Figure S4) it looks like it’s way more than 25 lines.
